# The impact of oil industry-related social exclusion on community wellbeing and health in African countries

**DOI:** 10.3389/fpubh.2022.858512

**Published:** 2022-10-19

**Authors:** Augusta C. Nkem, Stephanie M. Topp, Sue Devine, Wendy Wen Li, Daprim Samuel Ogaji

**Affiliations:** ^1^College of Public Health, Medical and Veterinary Sciences, James Cook University, Townsville, QLD, Australia; ^2^African Centre of Excellence in Public Health and Toxicological Research (ACE-PUTOR), University of Port Harcourt, Port Harcourt, Rivers, Nigeria

**Keywords:** oil industry, social exclusion, Africa, health, wellbeing

## Abstract

**Background:**

Oil is the mainstay revenue for a number of African countries. However, extraction can result in multiple impacts on the health and wellbeing of communities living in oil-rich areas. This review explored evidence of oil industry-related social exclusion on community health and wellbeing on the African continent.

**Methods:**

We used a systematic approach guided by PRISMA to search six databases for empirical and descriptive sources focused on oil industry impacts, in any African country, between 1960 to 2021. Findings were grouped according to four dimensions of the Social Exclusion Knowledge Network (SEKN) framework: political, social, economic, and cultural.

**Results:**

Fifteen articles were identified, of which 13 articles focused on Nigeria; while one focused on Sudan, and one on Côte d'Ivoire and South Africa. Evidence relating to political aspects of social exclusion encompassed marginalization of indigenous communities through land grabs and unequal representation in political decision making. Limited compensation for environmental damage and livelihood displacement caused by oil-extraction, and high rates of unemployment and poverty were key themes of the economic dimension. Evidence of social impacts included lack of government, or oil-industry investment in social infrastructure; poor health and wellbeing linked to land, air, and water pollution; homelessness and lack of social cohesion. The cultural dimension of social exclusion was comparatively underexplored and only six sources included data collection with indigenous residents, and comparatively more sources were written by non-citizens or non-residents of oil-industry affected areas. Major themes included impacts on collective identity, ways of life and values, particularly where loss of ownership or access to land was experienced.

**Conclusion:**

Oil industry activities in African countries are clearly associated with multiple exclusionary impacts. However, the narrow body of empirical research limits understanding of the lived experiences and management of social exclusion by residents of oil-rich areas themselves and is an area deserving of further attention.

## Introduction

Oil is among the most impactful of the various extractive industries globally and in Africa. In 2020, 10 African countries were identified as being the top oil producers (Nigeria, Algeria, Angola, Liberia, Libya, Republic of Congo, Ghana, Gabon, Equatorial Guinea, and Chad) despite (1). Five African countries in particular – Nigeria, Angola, Liberia, Libya, and Egypt – accounted for ~9.6% of the world's output with 7.9 million barrels per day, although this is a below the production levels between 2005 and 2010 (2). Despite a growing number of alternative energy sources globally, oil production has played an important role in supporting nations whose governments are heavily reliant on oil income.

Landowners and residents of natural resources rich countries have historically been excluded from sharing in the revenue and other benefits generated from resource extraction (3, 4). A well-established pattern in which oil corporations and governments exploit resources without involving or compensating residents and landowners is recognized (5–7) with landowners excluded from oil revenue (8, 9) and lack of compensation for, or reinvestment of oil resources into “host” communities (3, 8, 10, 11).

Negative impacts relating to environmental damage and health are well recognized. For example, an incomplete record of oil spills in the Niger Delta Region (NDR) of Nigeria over the past 30 years shows that four million barrels of oil spilled between 1991 and 2011 and a further 12,381 spilled between 2011 and 2019 (12, 13). Alongside land clearing, gas flaring and infrastructure development, spills have left land and water ways inaccessible and unproductive (12, 13), resulting in further deforestation and exploitation of marginal land as people are pushed to find alternative sources of income (10, 14). Approximately 64% of the population of the oil-rich Niger Delta Region have neither a stable income nor access to basic amenities despite the oil resources deposited in the region (13–15) and in 2006, the United Nations Development Programme (UNDP) reported that the Niger Delta region's human development index was substantially below countries or regions with similar gas and oil reserves such as Venezuela and Indonesia (16).

Resource extraction activities degrade the environment, reducing residents' ability to farm, fish and live in an unharmed environment (8, 17). Loss of livelihoods contribute to poverty, undermining residents' ability to send their children to school or participate in skills building, further embedding this lack of opportunities (18, 19). Spills contaminate surface water, ground water, ambient air and crops with hydrocarbons, including carcinogens that are bio-accumulated in some food crops (20). Oil spills could lead to a 60% reduction in household food security as well as reducing the ascorbic acid content of vegetables by as much as 36% and the crude protein content of cassava by 40% (21). These reductions in food value could result in a 24% increase in the prevalence of childhood malnutrition (20). Studies have also linked industry-related pollution of land, air and water to tens of thousands of infant deaths annually, as well as heightened risk of kidney damage, cancer, diabetes, Alzheimer's and Parkinson's Disease (22). Loss of access to land may also impact on social cohesion affecting residents' opportunities to engage in traditional practices, compounded by disenfranchisement and lack of representation in the decision making that determines access to other resources (23).

### Conceptualizing the oil-industry impacts on resident communities

While coined a “resource curse” by some, oil industry impacts may also be conceptualized as *social exclusion* (24). Social exclusion has been defined as a deliberate act of depriving people of the opportunity to participate in social, economic, political, and cultural aspects of life (23), which impacts on individual and community wellbeing (25).

**Figure 1:**
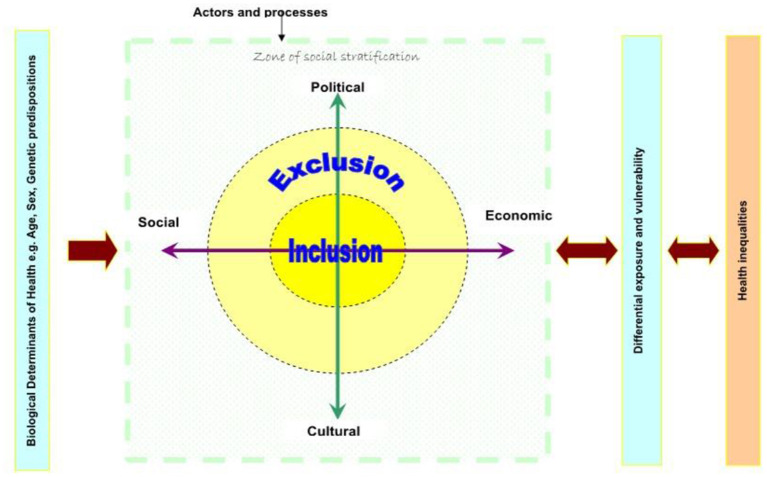
Social Exclusion Knowledge Network (SEKN). Adapted from World Health Organization (27); Understanding and tackling social exclusion.

Various frameworks have been developed to explore social exclusion in different settings (26) including the Social Exclusion knowledge network (SEKN) framework (27) (Figure 1). This framework uses a relational approach that defines social exclusion as a dynamic, multi-dimensional process and unequal relationship (27). The SEKN builds understanding of social exclusion from the influence of agents on four dimensions of social exclusion (political, social, economic, and cultural) that result in favorable or unfavorable outcomes for the actors who make up the social system. These exclusionary processes exist within a social system (family, household, nations, and global regions) and are underpinned by biological factors (age, sex, and genetic disposition). Interactions occur among the four relational dimensions of social exclusion and are influenced by systems of social stratification; that is, the way people are ranked and ordered in society based on factors such as level of education, occupation, income, and wealth. The level of stratification determines the level of access to resources, and ability to reduce exposure to health-damaging rights. Thus, low level stratification likely increases exposure to vulnerability, impacting health and wellbeing and embedding inequalities (27).

Research focused on oil industry-related social exclusion and its impacts on community health and wellbeing on the African continent has evolved during the past 15 years. Recognition of the way such social determinants, both individually and through interactions with each other, influence long term individual and community health and wellbeing is growing. However, mapping the depth and breadth of the evidence base is critical to understanding whether and where knowledge gaps still exist. Using a systematic scoping methodology, this paper investigates the state of knowledge regarding the impact of oil industry-related social exclusion on community health and wellbeing in sub-Saharan African countries. To the best of our knowledge, this is the first scoping review on impacts of oil industry-related social exclusion in Africa and first to apply the SEKN to explore oil industry social exclusion in Africa.

## Methods

A scoping review was conducted systematically using the Preferred Reporting Items for Systematic Reviews and Meta-Analyses (PRISMA) methodology and was guided by the six steps of the Arksey and O'Malley Framework for conducting a scoping review. This systematic process included identifying research questions, identifying, and selecting all relevant studies, charting the relevant data, collating, summarizing, and reporting the results (28). This approach enables researchers to thoroughly map the available evidence to discover gaps and report key concepts, location, time, and origin (28).

### Search strategy and selection criteria

Six electronic databases were searched for both peer-reviewed papers and gray literature: Scopus, Psyinfo, Pubmed, Medline, Proquest platform and Web of Science. In consultation with a university librarian, search terms were refined, and a search strategy devised that included four rows of search terms focusing on “oil industry” and like terms; African countries; “social exclusion” and like terms, and “community wellbeing” and like terms (full search strings appear in Appendix 1).

Inclusion and exclusion criteria were developed to select the relevant studies for this review. Literature was excluded if the primary focus was oil-related conflict, as conflict was conceptualized as an outcome of social exclusion, rather than a standalone dimension. Publications from 1960 to 2021 were included. The start date of 1960 was set because the modern commercial oil extraction activities began in Nigeria in the late 1950s. Table 1 summarizes the inclusion and exclusion criteria.

**Table 1:** Inclusion/exclusion criteria.

**Inclusion**	**Exclusion**
Literature examining or commenting on oil industry impacts on political, economic, social, or cultural aspects of resident populations in any country in Africa	Literature where one or more dimensions of social exclusion are mentioned but are not the focus of the article. Literature where the primary focus was conflict in oil-rich areas (*with conflict conceptualized as an outcome rather than driver of social exclusion*)
Qualitative and quantitative peer reviewed journals, theses, and dissertations	Expert commentaries and literature reviews
Only texts written in English	
Publications from 1 January 1960 to 31 May 2021	

A total of 3,274 records was identified, of which 617 were duplicates (617 duplicates were literature that appeared twice in the search result. Following PRISMA guidelines, the duplicates are recorded and removed). Following title and abstract review and application of inclusion and exclusion criteria, 51 full text articles were assessed, and 8 studies met the inclusion criteria. Following manual scanning of reference list seven additional records were identified. A total of 15 records are included in the final review.

**Figure 2:**
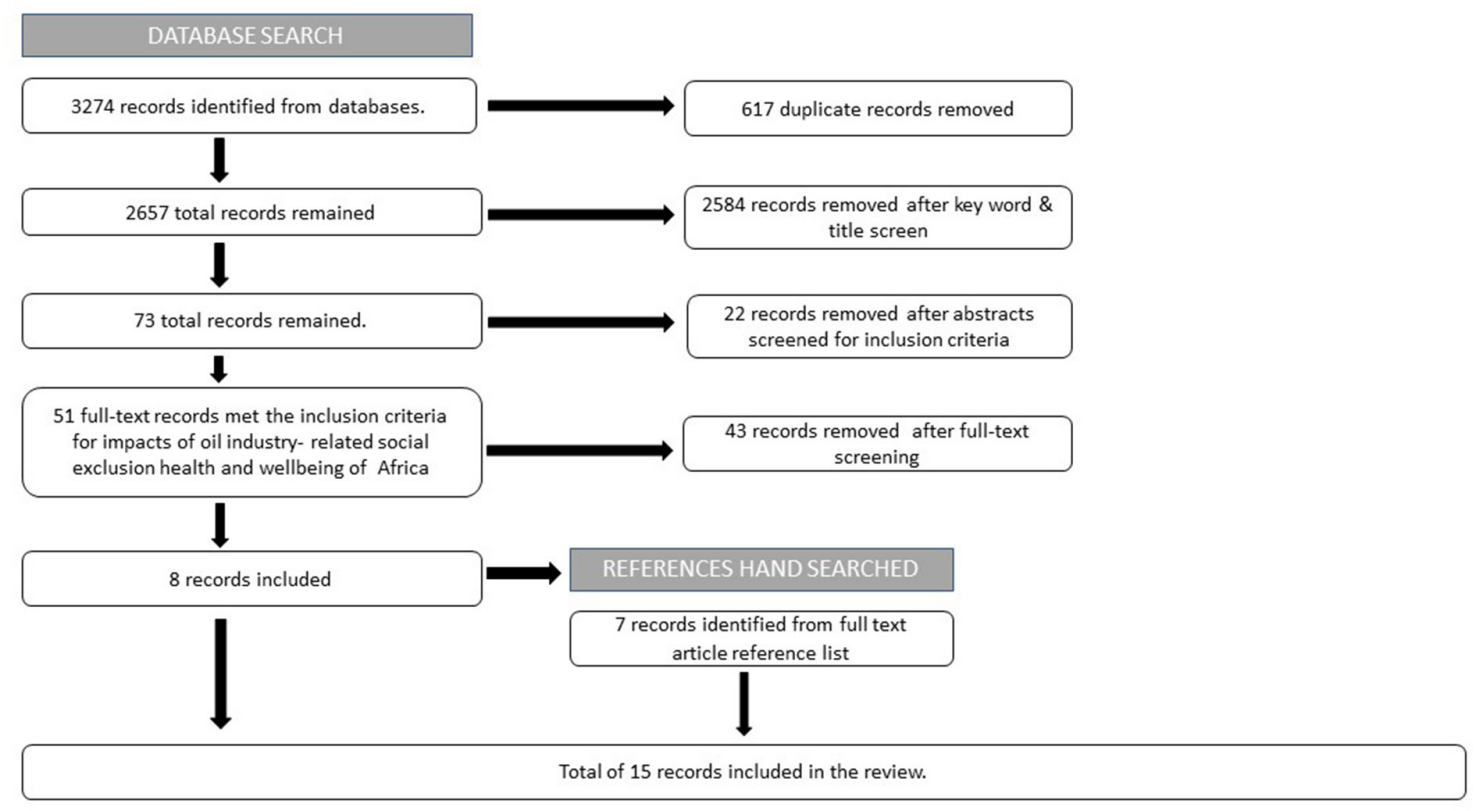
Prisma flow diagram.

The identification and screening process is summarized in Figure 2.

### Data extraction and analysis

All works, except for theses and dissertations (*n* = 6) were read in full by the first author; theses and dissertations were evaluated, and specific chapters selected and read based on their relevance to the review topic. Categorization of data in each dimension was initially based on examples of previous applications of the SEKN framework and iteratively adapted in consultation with co-authors as data extraction progressed. Natural overlap in some areas of impact (e.g., health impacts) were discussed and assigned based on consensus. The details of each of the 15 selected studies are summarized in Table 2 under the following headings: (i) Author, year of publication and journal, (ii) Title, (iii) Study location, (iv) Aim of study, (v) Study design and data collection methods, and (vi) Themes related to the dimensions of the SEKN.

**Table 2:** Included sources reflecting oil industry-related social exclusion in sub-Saharan African countries.

**Author/date**	**Title**	**Journal/Publication name**	**Study location**	**Aim of study**	**Study design and method**	**Summary data on social exclusion relating to political, economic, social and cultural domains**
1. Akpan (29)	Oil, people and the environment: Understanding land-related controversies in Nigeria's oil region	Journal of the Council for the Development of Social Science Research in Africa (CODESRIA) Rethinking African Development: Beyond Impasse, Toward Alternatives, Maputo. 2015/12	Nigeria	Aims to examine the causes of controversies surrounding land acquisition, compensation, and cooperation of the oil communities.	Qualitative empirical Participants are from Oloibiri, Ebubu and Iko (in Bayelsa, Rivers and Akwa Ibom states respectively)	Political The Nigerian government set up law enabling total ownership of land and the resources which neglects the original owners of the land Cultural Land appropriation without adequate cooperation from the communities
2. Edokpayi (30)	Shell Oil Company and Social Justice in the Niger Delta: The case of Shell in Ogoni, Nigeria	Master Thesis Published by Morgan State University, USA Publication number 1424540	Nigeria	Aim to examine the contribution of oil revenue to the Nigerian economy and evaluate the impact of Shell's operations in Ogoni	Empirical case study	Social Nigerian government and oil industries do not provide critical social amenities in Niger Delta Political Niger Delta being ethnic minority lacks political power to participate fully in making favorable decision-making on oil revenue sharing Economic The oil industry and Nigeria government exploit oil wealth in Niger Delta and exclude the people of Niger Delta from the revenue generated
3. Brown and Ogedengbe (31)	Compulsory acquisition of oil exploration fields in Delta State, Nigeria	Journal of Property Investment & Finance Volume 25 Issue 1	Nigeria	Aims to explore how compensation for compulsory acquisition of land for oil activities works in the Niger Delta region	Quantitative Survey questionnaires	Cultural Land in Niger Delta is forcefully acquired by the Nigerian government and the oil industry without adequate compensation to the traditional owners
4. Ogula (32)	Stakeholder involvement in corporate social strategy: An ethnographic study of the Niger Delta, Nigeria	Doctoral Dissertation Published by University of Phoenix, USA. Publication number 3345054	Nigeria	Aims to explore the perception of community members on corporate social strategy to understand the factors that influenced their perceptions.	Qualitative cross-sectional study One-on-one, interviews. Participants are Niger Delta inhabitants	Social Niger Delta Region lacks basic social amenities despite the massive oil revenue generated Cultural Oil industry destroys the land and water neglecting the need of the land and river by residents Political Nigerian government centralized the control of oil resources and exclude the residents of Niger Delta from equal sharing of revenue
5. Amaefule (33)	External Costs of Oil and Gas Exploration in the Niger Delta Region of Nigeria	Doctoral Dissertation Published by Argosy University Washington, DC	Nigeria	Aims to Investigates the phenomenal impact of oil and gas exploration in Niger Delta Region of Nigeria	Quantitative Survey methods with university students	Social Pollution from the oil industry contaminates the water and air causing emergence of diseases in Niger Delta Economic The employment opportunities are not commensurate to the entire loss of occupation by the residents of oil communities Political The Nigerian government does not involve the people of Niger Delta in making decisions regarding oil resources Cultural The occupants of oil communities are displaced from homes with the oil extraction on the land.
6. Geo-JaJa (25)	“Social Exclusion, Poverty, and Educational Inequity in the Niger Delta Region of Nigeria” in *The Politics of Education Reforms*	Book Chapter: “Globalization, Comparative Education and Policy Research” Chapter 7 Pg 111–135 Editors •Joseph Zajda •Macleans A. Geo-JaJa	Nigeria	Aims to discuss the problems involved with building social and economic assets in the Niger Delta	Case Study design, qualitative methods	Economic The Nigeria government does not share oil revenue equally to the Niger Delta Social There is lack of critical social amenities in Niger Delta Political The power structure in Nigeria affects Niger Delta as a minority ethnic group in decision making
7. Umejesi (34)	Land use, compensational justice and energy resource Extraction in Nigeria: A Socio-Historical Study of Petroleum and Coal Mining Communities	Doctoral Dissertation Published by University of Fort Hare, South Africa	Nigeria	Aims to examine the level of experiences and perceptions of compensation paid to landowners in Niger Delta region by oil industry on land acquisition	Qualitative and quantitative Observation, focus group discussion, key informant interviews and small-scale survey. Participants include community leaders, three elders, women leaders, youth leaders and staff of oil company	Social Massive land appropriation by the oil industry resulting to displacement of people and loss of communal connection in oil communities Economic Loss of farming and fishing occupation in Niger Delta Region resulting to loss of livelihood Cultural Loss of traditional ties to land and communal values because of mining and oil extraction Political Nigerian government enacted the Land Use Act of 1978 enabling the total ownership of the oil resources
8. Eserifa (35)	Holistic management: A conceptual framework in evolving sustainable corporate social responsibility	Doctoral dissertation Published by University, Phoenix, USA	Nigeria	Aims to explore the gap existing between corporate social responsibility programs (CSR) of oil industry and the needs in Niger Delta Region	Qualitative and Quantitative cross-sectional The interview and survey involved indigenes and community leaders affected by oil exploration activities	Social Oil spills and gas flare affect the health and environment of the people of NDR causing diseases and displacement Economic The people of NDR experience low income and loss of livelihood causing a strife between them and the oil industry
9. Reynolds (36)	Oil and political stability in Côte d'Ivoire and South Africa	Masters Thesis Publication by Webster University (Publication number 1526212)	Africa	Aims to discuss how natural resources has contributed to political stability and instability in African countries	Qualitative Empirical case study	Political Dependence on oil revenue by the government and corruption resulting to political instability and economic variability Economic Unequal distribution of oil wealth by the government and oil industry in African oil communities
10. Umejesi (37)	Amnesty patriarchy and women: the missing gender voice in post conflict Niger Delta Region of Nigeria	Gender and Behavior, 2014. **12** (1): p. 6223–6237	Nigeria	Aims to examine government's amnesty policy and program toward women development and inclusion	Qualitative Empirical data was collected using oral interview, focus group discussion and observation	Economic The people of Niger Delta have few opportunities to gain employment and experience unfair wealth sharing from the Nigerian government
11. Wiwa (7)	Youth Coping Strategies Resulting from the Niger Delta Oil Crisis	Master Thesis Published by The University of Guelph, Canada	Nigeria	Aims to investigate how the youths in Niger Delta survive with deteriorating environmental, social, and political conditions of the region	Qualitative Observation and semi-structured interviews Participants are men and women in Niger Delta between 18 and 30 years of age	Political The Nigerian government depends solely on oil revenue while neglecting the NDR in decision making regarding oil revenue Economic Unequal opportunities and unfair wealth sharing have caused unemployment and poverty in the Niger Delta Region
12. Hennchen (38)	Royal Dutch Shell in Nigeria: Where Do Responsibilities End?	Journal of Business Ethics, 129 (1), 1–25	Nigeria	Aims to assess the level of corporate responsibilities of oil industry toward the host communities	Qualitative empirical Interviews with former NGO directors' academics consultants, community relations officers and oil industry staff	Social Oil industry showing inadequate social and corporate responsibilities toward oil pollution Political Nigerian government is ranked among the most corrupt government due to the oil revenue embezzlement Economic Oil communities excluded from the enormous oil resources generated by the oil industry and the Nigerian government
13. Oduaran (14)	Effects of Petroleum Oil Spillage on Traditional Fish Farming in The Niger Delta	Master Thesis Published by Southern Illinois university at Edwardsville (Publication number 10192130)	Nigeria	Aims to examine the effects of oil industry extraction spill on fishing and fishermen income in Niger Delta Region	Quantitative Structured questionnaire survey The interview involved five key oil and gas managers	Social Petroleum oil pollution on the Niger-Delta has resulted to loss of fishing activities and the fishermen experiencing loss of communal connection Economic Oil spill contaminates fishing zone in Niger Delta Region causing loss of income to the Fishermen
14. Brino (39)	The Responsibility to Prevent: Neo-colonialism, Poverty and Mass Atrocity Crimes in Africa	Doctoral Dissertation Published by State University of New York at Albany (Publication number 10812518)	Sudan	Aims to discuss how land in Sudan was acquired by the British for oil extraction and for growing of cotton without recognizing the landowners needs	Qualitative Empirical case study	Social Extracting of natural resources without approval from the owners violates human rights of the people of Niger Delta Region Cultural The Sudanese government and the oil industry do not consider the land values and needs to the land to the original landowners Political The Sudanese government made policies that enables the government and the multinationals to exploit oil in Darfur region. Economic Neo-colonialism allows former colonies to maintain the economic benefits of colonialism through access to colony's natural resources
15. Habiba (40)	Conflicts in the Niger Delta: Analysis of Causes, Impacts and Resolution Strategies	Doctoral Dissertation Published by Coventry University, UK	Nigeria	Aims to investigate the causes, impacts of oil conflict and evaluate the resolution strategies in Niger Delta Region	Qualitative Grounded theory, and survey	Political The Nigerian government uses policies to gain total control of oil resources and deny the indigenous communities from benefitting from oil wealth Economic The residents of oil communities are marginalized from a fair sharing of the oil revenue

## Results

Fifteen articles were included in this scoping review. Among the articles that met the inclusion criteria were seven published between 2000 and 2010 and eight between 2011 and 2021. Despite rapid expansion of oil industry activity in the NDR from 1958 onwards, we found no empirical works exploring social exclusion (or related issues) in Nigeria or elsewhere in Africa between 1960 and 2004.

Of the articles identified, 13 of the total of 15 focused on Nigeria (7, 14, 25, 29–35, 37, 38, 40) while two articles focused on other African countries including Sudan (39), Côte d'Ivoire and South Africa (36).

Nine studies used qualitative methods (7, 25, 29, 30, 32, 36–39) two studies used mixed methods (34, 35) and three studies used quantitative methodology (14, 31, 33). All studies focused on at least one of the SEKN dimensions, while some focused on multiple dimensions. A majority (*n* = 10 of 15) discussed political aspects; more than half the sources also mentioned discussed economic dimensions (*n* = 11) and social dimensions (*n* = 9) while comparatively fewer sources identified cultural dimensions of social exclusion (*n* = 6).

### The political dimension of social exclusion

Eleven sources in this review focused on the political dimension of social exclusion). Empirical works included theses and dissertations (*n* = 8) and published peer reviewed papers (*n* = 3). Nine of the empirical works used qualitative methods and two used mixed methods. Most empirical works (*n* = 10) were focused on Nigeria (7, 25, 29, 30, 32–34, 38–40) with just one focused on South Africa and Cote I'voire (36).

The political dimension of social exclusion covers lack of negotiation power, political marginalization, and unequal power representation. The major themes in the reviewed literature were indirect rule of colonial masters; political power gaps and marginalization; over-dependence of oil revenue; corruption of the federal government, ethnic minority of original oil owners and exclusion in political decision making.

Several sources examined political dimensions of social exclusion through discussion of the way power dynamics in the Niger Delta region of Nigeria causes disparities in individuals' and groups' ability to access political rights through full participation. Brino (39) found that the former colonial powers in Africa enacted neo-colonial policies which indirectly controlled the political state of the colonies through their multinational oil companies. In Sudan, the exercise of power over oil resources and oil revenue to the exclusion of local populations was started by the former colonial power and effectively continued through a multinational oil company (Chevron), enabled by the Sudanese government (39). In Nigeria, Umejesi (34) outlined how the Nigerian Land Use Act 1978 enabled the federal government to deny the rights of traditional landowners and compulsorily acquire land for exploitation by multinational oil companies. Akpan (29), Amaefule (33), and Ogula (32)also highlighted the exercise of power over Niger Delta communities by the federal government of Nigeria and multi-national oil companies through legislative (e.g., 1978 Land Use Act) and political means, depriving traditional landowners access to their land or participation in decisions regarding oil deposits in the land. Hennchen (38) reports the Nigerian government is ranked among the most corrupt government linking this to revenue from oil.

A key aspect of social exclusion was the use of political power to control oil resources in ways that marginalized landowners. Key mechanisms included not recognizing individuals' or communities' original ownership, thus enabling land grabs Umejesi (34) and limited acknowledgment or recompense for the negative consequences of resource extraction on residents. In Nigeria, Habiba (40) highlighted the effects of oil extraction in communities where oil spills contaminate the environment with the federal government unwilling to hold multinational corporations to account. As a result of the environmental degradation, some communities actively engage in protests (40). Wiwa (7) noted the ongoing protests by communities in the Niger Delta region have been met with military force by the federal government, which has led to a number of deaths.

A number of sources note the ongoing exclusionary impacts of oil dependence. Geo-JaJa (25) and Habiba (40) discussed how in Nigeria the federal government's interest in oil revenue has directly impacted its willingness and ability to protect communities in oil-rich regions. Wiwa (7) described how the federal government of Nigeria has continually decreased the revenue derivation of the oil communities, enriching the political classes. This situation is exacerbated by an exploitation of Nigeria's federal political system which is linked to proportionate representation. Wiwa (7) observed the comparatively small size of the population in states of the Niger Delta region influences the number of representatives in parliament, and thus their voice in federal government. Wiwa (7), Geo-JaJa (25), and Edokpayi (30) each showed that this “minority status” places communities of the Niger Delta region at a disadvantage when it comes to political decision-making as the federal government does not consult the oil communities regarding oil projects and makes policies that are unsuitable to the oil communities. According to Geo-JaJa (25) the residents of oil communities should be involved in deciding social development significant to their wellbeing and participation in decision making provides them sense of inclusion. In Cote d'Ivoire, Reynolds (36) found that dependency of oil revenue by the Cote d'Ivoire government also led to corruption and affected the political stability of the country.

### The economic dimension of social exclusion

Eleven sources in this review identified aspects of the economic dimension of social exclusion. Most of the empirical works (*n* = 11 of 12) were focused on Nigeria (7, 14, 25, 30, 32–35, 38–40) with just one focused on Cote I'voire and South Africa (36).

The economic dimension of social exclusion relates to daily livelihoods; poverty; unequal wealth sharing, low income, non-availability, and accessibility of employment to the resource owners. Key themes discussed in relation to the economic dimension of social exclusion include loss of livelihood, loss of traditional occupation and low income due to the environmental effects of oil extraction, unfair oil wealth sharing, unemployment, and inadequate compensation.

Oduaran (14) and Umejesi (34) both demonstrated that the economic dimension of social exclusion manifests in the daily livelihoods of the communities in oil rich regions in Africa whose original occupation of farming and fishing are lost as a result of oil pollution on the environment. Similarly Oduaran (14) and Umejesi (34) observed that loss of traditional fishing and farming in Niger Delta region has affected the livelihoods of the fishermen and farmers who are unable to meet basic daily needs including inability to pay when necessary for medical needs. Amaefule (33) and Umejesi (37) found the employment opportunities being offered by the multinationals to the residents of oil rich regions were inadequate in the context of the high rate of unemployment caused by displacement and damaged livelihoods.

A number of works discussed the exclusion of oil communities from any direct benefits flowing from the oil revenue. Reynolds (36) revealed that the former colonies of oil rich African countries continue to benefit from the colony's natural resources. Geo-JaJa (25) and Hennchen (38) highlight the significant revenue generated from oil resources and shared by multinational corporations and the governments. Geo-JaJa (25), Edokpayi (30), and Eserifa (35) contrast these revenues with the relative impoverishment of the residents of oil communities. Despite the massive, accrued revenue, Geo-JaJa (25) all highlighted a high level of poverty in the Niger Delta region which is rated one of the least developed regions in the world.

Wiwa (7), Habiba (40), Brino (39) specifically described the unequal distribution of oil revenue as a contributory factor to low income among the residents of oil communities in Nigeria. Although, the oil industry has claimed to pay compensation to the communities, landowners have described this as insufficient when compared to the revenue generated from the communities (7).

### The social dimension of social exclusion

Nine sources in this review identified aspects of the social dimension. Empirical works included theses and dissertations (*n* = 6) and published peer reviewed papers (*n* = 3). Three of the empirical works used mixed methods and six used qualitative methods. Most of the empirical works (*n* = 8) focused on Nigeria (14, 25, 30, 32–35) with one focused on Sudan (39).

The social dimension of social exclusion includes the health impacts of polluting activities, lack of social infrastructure, homelessness, marginalization, lack of social cohesion and conflict. Eserifa (35) highlighted that spills and flares from oil industry pollute the environment affecting the health and wellbeing of the residents through air- and water-borne diseases (33, 35, 38). Ogula (32) found that oil and other mineral extraction in Africa comes with environmental degradation that includes contamination of drinking water with both short- and long-term health consequences. Several sources framed these impacts of environmental pollution as a denial of residents' right to health. In Sudan, for example, Brino (39) found that the right of natural resource owners was being violated in the process of extracting the resources by the Sudanese government.

Exacerbating the earlier-mentioned economic impacts of oil extraction, a number of sources noted the absence of government or industry investment in social amenities to ameliorate the effects of land grabs or pollution. In relation to Nigeria, Geo-JaJa (25), Edokpayi (30), Hennchen (38), and Ogula (32) all discuss the lack of investment by the federal government of Nigeria and multinational oil companies in establishing or maintaining basic social amenities such as hospitals, educational facilities, piped water, and toilet facilities in the oil-rich Niger Delta region. According to Geo-JaJa (25), Niger Delta residents are deprived basic amenities that could improve the health and productivity in life such as educational and health services.

The social and cultural impacts (see following section) include lack of basic social infrastructure reduces communities' ability to engage in traditional social activities such as team fishing and family farming (30, 32, 33). Strong social connections promote positive living and when those connections are damaged, the bond between people can deteriorate affecting lives and health. According to Eserifa (35) land acquisition and environmental degradation associated with the oil extraction has displaced many families and communities from homes, rivers, and farmlands, affecting the communal life and social connection (14, 34).

### The cultural dimension of social exclusion

Six sources in this review revealed cultural dimensions of social exclusion. Studies included theses and dissertations (*n* = 3) and published peer reviewed papers (*n* = 3). Four of the empirical works used qualitative methods and two used mixed methods. Most of the empirical works focused on Nigeria (29, 31–34) with just one focused on Sudan (39).

The cultural dimension of social exclusion considers the oil industry's exclusionary impacts on peoples' ways of life, beliefs, and values. In this review, major themes in the cultural dimension included loss of land due to compulsory acquisition and associated impacts on collective identity, ways of life and values. Brino (39) and Brown and Ogedengbe (31) observe that the forceful acquisition of land by national governments largely neglect the cultural (as well as economic) significance of land for traditional owners. In Sudan for instance, Brino (39) notes that lands were forcefully acquired with little consideration for the original occupants who were culturally connected to the land.

In Nigeria, the provisions of the Land Use Act 1978 dramatically changed Niger Delta indigenous communities' access to their lands almost overnight (29) with significant implications for identity and culture (34). Akpan (29) notes that the region was famous for a farming and fishing culture. Also, Ogula (32) and Umejesi (34) describe the land as the basis for many traditional occupations. After the introduction of the act, generations in the Niger Delta region have been denied opportunities to inherited traditional fishing and farming skills and practices from their parents due to lack of access to rivers and land (33). Moreover, since fishing and farming skills along with land ownership are seen as critical components of an individual or family's standing in community, lack of access to land and water have impacted culturally embedded concepts of identity and wellbeing. Beyond the obvious economic (livelihoods) and social (including health) impacts of such displacement, Umejesi (34) and Brino (39) note the cultural impacts of damaged collective identity, atrophied livelihood skills, access to traditional foods, and traditional values. Ogula (32) found that scarce land in the Niger Delta because of forced acquisition, is a significant factor in the conflict in the region.

Umejesi (34) noted that the family-owned farms and raffia palms which are central the identity of the Niger Delta region are lost as the oil industry grabs the land. Sacred ancestral shrines and cemeteries have been desecrated and destroyed by the oil industry in laying of oil pipes (29). In Ngwo of Enugu State in Nigeria, where sacred land is grabbed for mining, the residents believe that desecration of the shrines causes the gods of the land to stop increasing the crop yield of the community (34).

Similarly, in Sudan, the residents see land as their identity and depend on land for survival. Brino (39) notes that land has been forcefully appropriated in Sudan damaging residents' traditional links to land in addition to their basic livelihoods. Brino (39) further notes the impacts of the introduction and imposition of French language as an official language in Sudanese communities ignoring traditional language.

## Discussion

This scoping review aimed to scope the existing evidence regarding the impacts of oil-industry related social exclusion on health and wellbeing of residents of oil communities in Africa. Considering the large size of the oil industry, the limited literature that was identified was surprising. Only 15 studies spanning five decades of oil extraction in Africa were identified with no studies addressing oil-related social exclusion prior to 1999. To the authors' knowledge, this is the first to apply Social exclusion knowledge network (SEKN) framework to explore oil industry social exclusion in Africa.

Although the empirical literature identified was not as extensive as expected, sources identified clearly pointed to significant social, economic, political and cultural social exclusion impacts of the oil industry in African countries, but Nigeria in particular. Most of the empirical literature in this review (13 of 15 sources) focused on Nigeria and the Niger Delta Region with one additional source focused on multiple countries including Nigeria. This reflects, in part, the longer history of oil-related industry activities in that country as well as the attention that local conflict over oil industry presence has garnered in recent years. Indeed, at the screening stage of this review we found many articles focused on describing oil-related civil conflict and violence in the Niger Delta region (41). Concerns around oil-industry related social exclusion and the “resource curse” have been heightened in other African countries by the Niger Delta region experiences (8, 11, 42).

Overall, the review highlighted multiple aspects of political, economic, social and cultural exclusion that interact and influence each other to the detriment of local communities. For example, in the political dimension, articles reported how political marginalization and unequal representation of community interests in the Niger Delta region, interacted with unequal wealth sharing and loss of livelihoods in the economic dimension to exacerbate the exclusion of local communities (32, 34, 39). Also, lack of investment in social infrastructure by governments or industry and the environmental impacts of polluting activities contributed to aspects of cultural exclusion including reducing Indigenous populations' access to traditional farming land, fishing grounds and sacred sites.

Notwithstanding the evidence of political, social and economic forms of social exclusion, this review revealed that there is still limited empirical work on the topic of social exclusion given the long history of oil extraction on the continent, and in particular, limited research that centers the lived experiences of residents or communities in oil-rich regions. A range of non-empirical sources (e.g. commentary, desk review or opinion) not included in the formal review (6, 8, 10, 11, 17, 43–45) may make the evidence base on social exclusion in Africa appear larger than it is. Yet empirical work – particularly that drawing directly on residents' experiences – remain scarce. Only nine studies identified included data collection with Indigenous residents, most of which are dissertations or theses. This is particularly important for improving understanding of the *cultural* aspects of social exclusion, which were comparatively under-explored in the sources identified in this study (only six of 15 mentioned cultural dimensions). The review also highlights a need to conduct research that promotes a better understanding the links between cultural aspects of social exclusion and political, social and economic marginalization and of the different types of responses of residents to these experiences of social exclusion.

### Strengths and limitation

This review was conducted using thorough and systematic search approach with assistance from a research librarian. Deliberately inclusive date ranges were adopted to try to ensure all potentially relevant literature dating back to the inception of oil-industry activity in Africa was capture. Nonetheless, our primary reliance on academic data bases and restriction to English language sources means it is possible that some studies may not have been identified which could have added further depth of understanding to the topic being explored. Another limitation was the exclusion of literature specifically focused on oil conflict which prevented deeper reflection on the role of oil conflicts themselves on social exclusion and its implications. Furthermore, because of the extensive nature of gray literature, our gray literature search was necessarily targeted, and important sources kept in local repositories that the authors were unaware of may have been missed.

## Conclusion

Oil industry activities in African countries are clearly associated with multiple exclusionary impacts. This review provides evidence of how oil industry activities affect the political, social, economic, and cultural lives of oil communities' residents in Africa and impact on health and wellbeing. Exploring the perception of the residents toward oil industry-related social exclusion on political, social, economic, and cultural aspect of their lives would enhance the understanding of the impacts of social exclusion on the health and wellbeing of oil communities and thus, help to inform effective policies toward improving inclusion in oil communities in Africa. However, the narrow body of empirical research limits understanding of the lived experiences and management of social exclusion by residents of oil-rich areas themselves and is an area deserving of further attention.

## Data availability statement

The original contributions presented in the study are included in the article/Supplementary material, further inquiries can be directed to the corresponding author.

## Author contributions

AN, ST, and WL: conceptualized the study. AN: wrote first draft, data search, and extraction. AN, ST, and SD: data analysis. AN, SD, ST, and DO: critical edits. All authors approved final version.

## Funding

This study was conducted with support of a James Cook University Postgraduate Research Scholarship to (AN) and the College of Public Health, Medical and Veterinary Sciences (CPHMVS) Higher Degree by Research Enhancement Scheme (HDRES) Publication Grant.

## Conflict of interest

The authors declare that the research was conducted in the absence of any commercial or financial relationships that could be construed as a potential conflict of interest.

## Publisher's note

All claims expressed in this article are solely those of the authors and do not necessarily represent those of their affiliated organizations, or those of the publisher, the editors and the reviewers. Any product that may be evaluated in this article, or claim that may be made by its manufacturer, is not guaranteed or endorsed by the publisher.
