# Supplementary material for: The impact of oil industry-related social exclusion on community wellbeing and health in African countries
Source: Front Public Health. 2022 Oct 19;10:858512. doi: 10.3389/fpubh.2022.858512 (PMC9627145; doi:10.3389/fpubh.2022.858512)
Supplement: Supplementary file 1 [file Data_Sheet_1.pdf]

Rows	Key search terms
1	"oil industry" OR "oil industries" OR "petroleum industry" OR "petroleum industry" OR "petroleum mining" OR "oil extraction" OR "petroleum extraction” OR “oil company” OR “oil companies” “petroleum company” Or “petroleum companies” OR"agip" OR "shell" OR "LNG" OR "elf"
2	“Africa* OR Algeria OR Angola OR Benin OR Botswana OR "Burkina Faso" OR Burundi OR "Cabo Verde" OR Cameroon OR "Central African Republic" OR Chad OR Comoros OR "Democratic Republic of the Congo" OR "Republic of the Congo" OR "Cote d'Ivoire" OR Djibouti OR Egypt OR "Equatorial Guinea" OR Eritrea OR Eswatini OR Swaziland OR Ethiopia OR Gabon OR Gambia OR Ghana OR Guinea OR "Guinea-Bissau" OR Kenya OR Lesotho OR Liberia OR Libya OR Madagascar OR Malawi OR Mali OR Mauritania OR Mauritius OR Morocco OR Mozambique OR Namibia OR Niger OR Nigeria OR Rwanda OR "Sao Tome and Principe" OR Senegal OR Seychelles OR "Sierra Leone" OR Somalia OR "South Africa" OR "South Sudan" OR Sudan OR Tanzania OR Togo OR Tunisia OR Uganda OR Zambia OR Zimbabwe”
3	"Social isolation" OR "social exclusion" OR ostracism OR social exclusions OR marginal* OR "socially excluded" OR "socially included" OR "social deprivation" OR "socially deprived" OR "lack of contact" OR "Social Belonging" OR "social inclusion"
4	"Community wellbeing" OR "Wellbeing" OR "wellbeing" OR "health" OR "empowerment" OR "stress" OR "anxiety" "community" OR "collective" OR "communalism"
